# Understanding Education Workers’ Stressors after Lockdowns in Ontario, Canada: A Qualitative Study

**DOI:** 10.3390/ejihpe13050063

**Published:** 2023-05-09

**Authors:** Frances Serrano, Marianne Saragosa, Behdin Nowrouzi-Kia, Lynn Woodford, Jennifer Casole, Basem Gohar

**Affiliations:** 1 Department of Population Medicine, University of Guelph, Guelph, ON N1G 2W1, Canada; 2 Department of Psychology, University of Guelph, Guelph, ON N1G 2W1, Canada; 3 Lunenfeld-Tanenbaum Research Institute, Sinai Health System, Toronto, ON M5G 1X5, Canada; 4 Department of Occupational Science & Occupational Therapy, University of Toronto, Toronto, ON M5G 1V7, Canada; 5 Centre for Research in Occupational Safety & Health, Sudbury, ON P3E 2C6, Canada; 6 Insight Psychology on Norfolk, Guelph, ON N1H 4J4, Canada; 7 Department of Special Education, Loretto College, Toronto, ON M6H 2N1, Canada

**Keywords:** COVID-19, K-12 education, online teaching, remote learning, Canada

## Abstract

Understanding the experiences and stressors of education workers is critical for making improvements and planning for future emergency situations. Province-specific studies offer valuable information to understand the stressors of returning to the workplace. This study aims to identify the stressors education workers experienced when returning to work after months of school closures. This qualitative data is part of a larger study. Individuals completed a survey including a questionnaire and some open-ended questions in English and French. A total of 2349 respondents completed the qualitative portion of the survey, of which most were women (81%), approximately 44 years of age, and working as teachers (83.9%). The open-ended questions were analyzed using thematic analysis. Seven themes emerged from our analysis: (1) challenges with service provision and using technology; (2) disruption in work–life balance; (3) lack of clear communication and direction from the government and school administration; (4) fear of contracting the virus due to insufficient health/COVID-19 protocols; (5) increase in work demands; (6) various coping strategies to deal with the stressors of working during the COVID-19 pandemic; (7) lessons to be learned from working amid a global pandemic. Education workers have faced many challenges since returning to work. These findings demonstrate the need for improvements such as greater flexibility, training opportunities, support, and communication.

## 1. Introduction

The novel coronavirus disease COVID-19 has significantly changed how people live and work. Globally, governments mandated practices such as social distancing to minimize person-to-person physical contact and reduce the spread of COVID-19 [1]. As a result, educational institutions were forced to suspend in-person learning and shift to distance and online learning [2,3,4]. In Ontario, Canada, schools were closed physically in the spring of 2020 amid the first pandemic wave [5]. Like other occupational groups, the education workers were forced to make rapid changes in providing services to their students. Both elementary and high schools introduced emergency remote teaching (ERT) to accommodate social distancing restrictions [6,7], emphasizing the importance of information and communication technology (ICT) and a decrease in the importance of the physical location of work [8]. ERT refers to courses designed initially for in-person instruction that were modified for online instruction due to emergencies [7].

Among the challenges faced by education workers, particularly teachers, was the immediate need to teach virtually [9]. Before the pandemic, educators had limited online teaching experience using technologies within their classrooms [10]. Unsurprisingly, educators reported stress and anxiety while working irregular hours due to limited time to prepare and train for such an undertaking [10,11,12]. More time and effort planning lessons and adopting novel teaching strategies led to an increase in workloads [10]. Moreover, teachers need to communicate content through a screen, engage students using audio and visual materials, facilitate interactions and attend to student needs virtually, and troubleshoot technical problems [13]. This increase in workload was one of the most significant stressors during the pandemic [10]. Other stressors included unclear boundaries between work and home life, the stress of online teaching, and financial stress [11].

Education workers across the province of Ontario returned to work in the fall semester of 2020 for the first time since the first wave with strict COVID-19 safety protocols, while a COVID-19 vaccine had yet to be approved. Surveyed Canadian teachers reportedly welcomed their return to in-person learning [14]; however, despite their return to school, education workers continued to experience higher working demands with unrealistic expectations [14].

During the time of this study (Fall of 2020), schools were beginning to reopen after several months of teaching remotely, and education workers were returning to in-person classes. Concurrently, the second wave of COVID-19 was rapidly spreading. To reduce growing concerns over the return to work, the provincial government enforced rules limiting contact with others, and it began promoting ways of protecting oneself against the virus [15]. Social distancing measures, wearing masks, and disinfecting classrooms all became a part of the school environment, affecting the daily routines of students and teachers. Furthermore, for families who chose to keep their children home, education workers had to change to hybrid or blended models of teaching pupils. In other words, some students were present in the physical classroom while others were connected via virtual classrooms, and teachers were expected to instruct children in physical classrooms while supplementing the learning of students in virtual classrooms [16]. Unsurprisingly, lesson planning was labour-intensive, as teachers now had to accommodate for two classrooms. Although there was an increase in work demands, the amount of support or time given to complete these demands remained unchanged. High schools implemented a new schedule for students known as a “quadmester”. Students would be taught in one class all day each day for one entire week. The following week, they would take a second subject with a different cohort of students. These two courses would be completed in 9 to 10 weeks, at which time the students would start two new subjects [17].

Given the impact of the pandemic on education workers, identifying and understanding their stressors is important for future planning. This study’s primary objective was to identify and understand the specific stressors that education workers have been facing upon their return to school. The secondary objective was to identify information about working from home to help the province of Ontario prepare for future emergencies.

## 2. Materials and Methods

### 2.1. Design

This descriptive qualitative study is part of a larger mixed-method project examining education workers’ psychosocial well-being and functional ability [18] across Ontario during the pandemic. From October 2020 to January 2021, Ontario education workers were asked to voluntarily complete an anonymous survey that included questionnaires and open-ended questions. The survey was disseminated on behalf of the researchers by various educational unions at the elementary and secondary levels in English and French.

### 2.2. Participants

We operationalized an education worker as any unionized employee working in the public education system in Ontario, Canada. This definition includes various occupational groups who provide educational support, such as teachers and educational assistants. It includes professional support services such as child and youth counselling, psychology, social work, and speech services. It also includes non-student roles, including administrative jobs (e.g., office coordinators and information technology roles) and maintenance.

### 2.3. Data Collection

The questionnaire contained various demographic questions related to the respondents’ characteristics such as age, identified gender, marital status, dependants, geographical location and population density, and employment type (title, part-time vs. full-time, and permanent vs. contract). Additionally, the questionnaire included three open-ended questions. In the first question, we asked, “Without indicating identifiable information, please tell us the most stressful part of your job since returning to work during the COVID-19 pandemic.” In the second question, we asked, “Without indicating identifiable information, please tell us if there have been any good coping strategies to deal with working during the COVID-19 pandemic.” Finally, we asked, “Without indicating identifiable information, tell us any information that could help us (lessons learned) in case we are asked to work from home again.” French responses were translated before the analysis.

### 2.4. Data Analysis

We analyzed the demographic data using SPSS 28.0 for Mac [19]. For the open-text questions, we used an iterative approach in coding the data to ensure the data’s reliability. Common themes were identified from the open-text questions based on Braun and Clarke’s (2006) six steps of thematic analysis. First, data were read multiple times to gain familiarity [20]. Next, two authors (F.S., B.G.) developed codes inductively and collectively reviewed the codes for the data set. At this stage, coding differences were discussed and resolved by further refining the coding framework. Together, the coders identified common and meaningful aspects of the data, and repeating patterns were identified with specific codes. The codes were then clustered based on similar and parallel findings to construct themes and subthemes when applicable. Codes with similar content were grouped together to create broader themes and iteratively reviewed to ensure their applicability to the theme. Finally, we revised the themes, subthemes, and codes in relation to the entirety of the data set to ensure the analysis was germane to the study’s objectives. Related or overlapping themes and subthemes were combined, and themes, subthemes, or codes of low relevance were not included. F.S. and B.G. were the primary and secondary coders, respectively.

### 2.5. Ethical Considerations

The study was approved by the University of Guelph’s Research Ethics Board (REB# 20-06-002). Participants were informed about the nature of the study through a written description and informed consent document preceding the study’s questionnaire. All participants gave informed consent to participate and were told that participation was voluntary and that they could withdraw their consent to participate at any time.

## 3. Results

### 3.1. Demographics

A total of 4394 education workers opened the survey. About 80% of the respondents completed the questionnaire in English and 20% in French. Overall, the average age for the sample was approximately 45. Over 81% of the respondents identified as women, and almost 80% identified as Caucasian/White. Over three-quarters of the participants were married, either common-law or in a committed relationship, and over two-thirds had dependants at home. The sample was predominately teachers (over 87%), including special education, occasional, and substitute teachers (Table 1).

Almost 90% of the respondents worked permanently full-time and were evenly split between staff working in elementary and secondary settings. The Toronto region had the highest proportion of responses (11.2%), followed by Ottawa (9.3%), Waterloo (7.8%), and Peel (7.6%). About 77.7% worked in urban settings (i.e., a population greater than 10,000), 18.6% worked in rural small towns (population between 1000 and 9999), and 4.7% worked in rural areas (i.e., a population less than 1000).

Of those who attempted the survey, 54.5% (n = 2349) responded to the qualitative portion of the survey. Among the participants who answered the open text questions, 92% (n = 2166) completed all three questions. The responses varied in length and detail.

### 3.2. Themes

Seven core themes emerged from our analysis: (1) challenges with service provision and using technology due to lack of training and limited resources; (2) issues with separating work and private life during the work-from-home period, creating a disruption in work–life balance; (3) difficulties managing the return to physical classrooms resulting from a lack of clear communication and direction from the government and school administration; (4) fear of contracting the virus due to insufficient health/COVID-19 protocols; (5) increase in work demands following the return to physical classrooms and the implementation of the hybrid learning model and quadmesters; (6) various coping strategies developed to deal with the stressors of working during the COVID-19 pandemic; (7) lessons to be learned from working amid a global pandemic and improving the workplace in case of future emergencies (Table 2).

The aforementioned themes reflect participants’ experiences both during a work-from-home period and their return to the workplace for the first time since the first wave of the pandemic, which was around the second wave of the pandemic. It should be noted that terms such as “some” or “several” do not indicate quantification of the data but are used to describe the extent to which a theme/code was prominent across participants.

#### 3.2.1. Theme 1: Challenges with Service Provision and Using Technology Due to Lack of Training and Limited Resources

Our results revealed significant challenges with the use of technology. According to the participants, transitioning from traditional to technology-based teaching brought many challenges. Using and integrating technology in education was a major stressor, as education workers with little to no experience with technology were forced to learn new programs rapidly. As one participant described it:

*“A shift to distance instruction, even within the classroom, without any kind of support has made the job very difficult to accomplish and the students have suffered as a result.”*

Many participants expressed insufficient support or training provided by their schools during the rapid shift to online learning. Reportedly, the resources and information school administrators gave were often insufficient to move to online learning effectively. Education workers were forced to find their own digital tools for online teaching and to compensate for the lack of resources or support.

*“Technology and the lack of time for training. We have to do it ourselves in our time and it’s not easy if you are already experiencing challenges with technology.”*

Since returning to the classroom, education workers reported worrying about switching back to remote learning. They expressed their concerns about their future, as the unpredictability of the pandemic has affected the educational system.

*“The fear and worry of having to switch back to remote learning at the drop of a hat and the fear of having to do synchronous on line lessons. Also knowing that I have not been properly trained on things like Microsoft teams, etc… which we are expected to know how to do in the event of school closures.”*

#### 3.2.2. Theme 2: Issues with Separating Work and Private Life during the Work-From-Home Period, Creating A Disruption in Work–Life Balance

When schools were closed, education was moved into virtual classrooms, meaning most education workers worked from home. Consequently, this affected work–life balance, as the boundaries between work and personal life became obscured. The tasks and duties assigned to education workers were accessible from home. This may have led to them struggling to limit the amount of work they were doing, as they were forced to work and live in the same space.

*“Having to teach 8 subjects at the same time as 5 like last year. Working 80 h a week, neglecting my family, not having enough time to clean, thinking about me.”*

Education workers had difficulties adjusting to work demands while tending to the needs of their families. The increased workload affected work–life balance, as individuals struggled to complete their work while caring for family members. The lack of time to complete work during the day impacted families, as education workers reported working overtime to manage their workload.

*“I don’t have enough time to do my work during the day so most nights are spent doing work. It’s been really hard to balance work with my family life (spouse and children).”*

Several participants reported trying to regain a balance between work and private life by limiting work to only certain hours of the day. As one participant described it:

*“The biggest thing that helped me was turning off my email notifications from my phone- creating boundaries for when I answered emails to only during work hours. You have to separate work and home when it’s one and the same.”*

#### 3.2.3. Theme 3: Difficulties Managing the Return to Physical Classrooms Resulting from a Lack of Clear Communication and Direction from the Government and School Administration

The COVID-19 pandemic led to many changes affecting teachers and staff members. The government was making constant changes to help reduce the spread of COVID-19. Subsequently, the constant changes led to conflicting information being spread across schools. According to respondents, the government was making decisions that did not reflect the recommendations of Public Health, creating turmoil among the public.

*“There seems to be a lot of conflicting information that seems counter to that of Public Health.”*

Moreover, teachers were not told about important changes until the last minute, including instructions on how to proceed with their work. This perceived lack of communication from the government created frustration, as education workers could not get vital information necessary to complete their job.

*“I have been told that I teach online 3 days a week (and face-to-face the other days) one day before the first teaching day.”*

Education workers became responsible for keeping up to date with important policy changes as communication with leaders became unreliable.

*“Teachers and educators have not been engaged in any meaningful communication or discussion about how to proceed”*

Education workers were frustrated and felt unappreciated as the government and school boards made decisions without consulting employees. Many felt that their needs went unmet and that the decisions made on their behalf did not benefit them.

*“Superiors make general statements, and we are given no concrete directives about how to implement those into our specific areas. The statements are often not well thought out and have significant implications beyond what the intentions were. Classroom teachers are not included in the conversation to discuss potential concerns with pursuing a course of action.”*

#### 3.2.4. Theme 4: Fear of Contracting the Virus Due to Insufficient Health/COVID-19 Protocols

Reportedly, the return to work caused stress amongst education workers, as the health and safety of employees became a concern. The increased exposure to other individuals had affected the perceptions of safety, as fears of contracting the virus arose. Participants reported feeling worried about infecting members of their household. For example, one participant said:

*“The constant worry that I’m going to be forced to teach in an unsafe work environment in which I can catch a disease that I might pass to my family, is killing me slowly.”*

Although the government and schools imposed safety measures and guidelines to minimize the spread of COVID-19, these attempts at creating a safe working environment felt inadequate or inconsistent. Education workers reported school administrators’ lack of enforcement of the new COVID-19 rules and protocols. Moreover, additional measures protecting employees against the virus were scarce. Social distancing guidelines could not be fully followed, as students and teachers were put into classrooms that had not been modified to meet the COVID-19 standards. In addition, some classrooms had inadequate ventilation and windows. The poor air quality within these classrooms was a health concern for many individuals.

*“Failure to comply with public health instructions in schools. It is impossible to ensure a distance of 2 m and the classes have no ventilation and no windows.”*

Classrooms were commonly described as being filled over capacity and not large enough to keep children apart in accordance with COVID-19 guidelines. The physical size of classrooms and the number of students assigned to a classroom made it difficult to maintain social distancing. Social distancing rules required individuals to be at least 2 m away from each other, but education workers reportedly found this difficult to accomplish.

*“We require 2 m and yet with the number of students in our class, only 1 m is possible. Even during lunch hours without the masks. In addition, we have no designated place other than the corridor to have our dinner without being disturbed by the movement of students from other classes.”*

Furthermore, the non-compliance among staff and students to the COVID-19 protocols set in motion by the government and school administration increased the risk of infection and caused a growing fear of transmission.

Education workers reported that their colleagues and students did not share the same concerns over the virus and were jeopardizing the health of individuals around them. Some colleagues were not enforcing the COVID rules, and the use of masks and maintaining social distancing were not being followed. Respondents reported that students refused to follow the COVID-19 guidelines, as many dismissed the social distancing rules and kept close contact with others. The use of masks was enforced inside the classroom, but students did not wear these masks outside during recess.

*“Feeling like only some staff members are following safety protocols while others have no regard. Management is aware but doesn’t do anything about it.”*

*“All students enter and exit for recesses without masks and eat lunch and snacks within their classrooms without mask. Many class sizes are at max or even over cap. Students are not properly social distanced from each other or their teacher. within class. At recess there is no social distancing.”*

#### 3.2.5. Theme 5: Increase in Work Demands following the Return to Physical Classrooms and the Implementation of the Hybrid Learning Model and Quadmesters

Participants indicated that even during a period of uncertainty and uneasiness from a global pandemic, education workers were told to keep up with work demands and maintain the academic standards of the schools. When education workers returned to their physical classrooms, they were assigned tasks to keep the classroom safe and minimize the spread of the virus. In addition to their normal responsibilities, new COVID-19 guidelines made education workers responsible for disinfecting and sanitizing classrooms. All individuals had to strictly abide by these rules, which further increased work demands and workloads. Overall, the increase in work demands and workloads was greater than the amount of time given to complete tasks, making it challenging for participants to finish their work on time.

*“We are expected to do more with less time. This results in very long hours of work. I regularly work 12+ hours per day.”*

*“Keeping my work space clean, reminding students of the protocols, repeating instructions over and over, working in all the PPE, working online and in class at the same time, added responsibilities but no extra time to do it.”*

Education workers described the adjustment to hybrid learning as challenging. Using the hybrid learning model increased teachers’ workloads, as the preparation of lessons had to include content for students attending physical classes and virtual classes. Consequently, teachers were not always available to assist with online student learning, thereby forcing them to provide students with enough resources and information to facilitate independent learning. The lessons posted online needed alterations to fit the needs of online students while also maintaining the academic standards.

*“Teaching in hybrid is a NIGHTMARE. It’s like I have to prepare for two separate classes and teach differently to each group, so it’s like double prep sometimes. I feel like I just can’t keep up. I spend hours and hours prepping for the next day because of the different schedule with so much more time needed to fill in the one class (8:15–12:35). Then when I finish for the day, I go home and prep to do it all over again the next day.”*

In high schools, quadmester schedules were utilized in addition to the hybrid learning model. High school courses were condensed and made to fit a shorter timeline, increasing work demands for education workers. Quadmesters required extra planning to be integrated into classrooms, as teachers had to create longer lessons to cover more course content.

*“Managing multiple platforms of teaching (in-class, asynchronous, synchronous). Managing multiple sections with strange timetabling for the board’s quadmestering model.”*

#### 3.2.6. Theme 6: Various Coping Strategies Developed to Deal with the Stressors of Working during the COVID-19 Pandemic

To cope with the stressors of working during the pandemic, education workers adopted strategies to help reduce stress and maintain mental health. Many of these non-work-related activities are related to self-care and personal time, such as physical activities and meditation. Many individuals reported taking time off from work and having mental health days. During this time, individuals would focus on engaging in self-care practices and not worrying about their work obligations. Moreover, some individuals sought out professional help to manage the stress.

*“Short leave of absence to help break the stress and anxiety cycle and start treatment.”*

Another strategy commonly utilized was to engage in physical and mindfulness activities. This included exercising at the gym, taking walks outside, playing sports, and performing yoga and deep breathing exercises. These strategies helped participants cope with the stress by having them engage in relaxing activities and thinking positive thoughts.

*“I starting meditating and doing deep breathing exercises on my own time. Some days, it is the only thing that gets me through the day.”*

Other strategies focused on maintaining social relations with colleagues. Communicating with team members on how to handle certain situations helped to minimize the stress felt by participants. Open communication on issues affecting employees were addressed, and team members were working together to handle these situations.

*“Communicating openly with my department members to manage problems at they arise.”*

Similarly, collaborating and developing relationships with colleagues were beneficial for some education workers. The planning and preparation of lessons became easier for teachers who were working in collaboration with colleagues teaching the same subject and cohort. These individuals reported feeling less stressed when they had support from their colleagues who were experiencing similar stressors.

*“Working with colleagues to collaborate on planning/prepping and to vent to has been the best way to cope. I wouldn’t survive virtual teaching without my colleagues support.”*

#### 3.2.7. Theme 7: Lessons to Be Learned from Working Amid a Global Pandemic and Improving the Workplace in Case of Future Emergencies

Respondents offered suggestions for future crisis management and communication in education. Specifically, they suggested that online courses should be developed by the school administration and include premade lessons for teachers to use. Furthermore, consistency in the online delivery method is crucial for education workers, as it enables them to stay organized and give all students the information needed to succeed.

*“I think The biggest mistake we made was having teachers create their own lessons. There should be curated lists of programs and sites that students can access that we can mark, but not have to all be inventing the wheel. It was a colossal failure on the governments part not to have programs prepared. It created a huge equity of access piece and A LOT Of stress on Teachers. There are some awesome self directed learning programs out there (many that don’t that require subscriptions to track) that should be either supported by or curated by the government.”*

Additionally, education workers needed certain equipment to complete their work, but many did not have access to these materials. Education workers suggested having more resources and training available for all employees.

*“Teachers do not have the proper tech tools to do the job that is being asked of them. The school equipment cannot keep up and has become a limiting factor for getting work done. This is a cause of stress.”*

*“Training on online platforms would be an ideal start. No one has received ANY training on working remotely in any capacity. Many programs aren’t conducive to online learning (phys ed, tech classes, food and nutrition, etc.) and there has been little to no assistance with ensuring said programs can run effectively to ensure a robust learning environment for students.”*

Individual considerations mentioned by respondents included designating a space in the home for work-related purposes only. Setting boundaries is important to maintain a work–life balance. Outside of the workspace, the priority of the individual should be to engage in activities focused on self-care, such as hobbies, sports, and meditation.

*“Have a designated space for work and close the door to that space when you are not working. Create as much separation as possible.”*

Returning to traditional in-person teaching was not welcomed by all participants. Some participants suggested using a work-from-home arrangement during emergency teaching. In emergency crises such as COVID-19, they believe that an effective way of reducing the risk of infection is to limit physical contact with others and limit travel to necessary trips only. One participant said:

*“I appreciate being able to work from home and not having to increase my risk of catching COVID by being forced to be in-school. Reminding myself of how lucky I am to be able to work from home helps.”*

## 4. Discussion

Our study examined the perceptions of education workers following the return to school amid the COVID-19 pandemic. Education workers identified specific stressors that affected their return to work and reported coping strategies they used to manage these stressors. From the data, we outlined a set of recommendations for consideration in a future crisis management situation.

Since returning to in-person learning following the shift to remote teaching, education workers described the stressors that have impacted their working experiences. One stressor identified in this present study is the use of technology in place of traditional teaching methods. Due to the rapid shift to online learning, teachers could not receive adequate training on the use of technology in the classroom. As a result, the transition to remote learning was difficult, as education workers felt that they did not receive enough support in using technology. Our findings echo Abilleira et al.’s (2021) report on the lack of instructions from the organization and feelings of techno-inefficacy experienced by teachers [21]. Feelings of inadequacy using digital tools were cited as a reason behind the decline in job performance [21]. Our findings support the argument that educators lack confidence in their ICT skills. The COVID-19 pandemic forced schools to adopt online teaching methods without providing workers with adequate ICT training to engage effectively in online teaching [3,22]. Education workers have reported having difficulties using ICTs, such as for accessing media and digital resources [23].

Our findings suggest that education workers are worried about their health and safety, their students, and their families. These results are consistent with a recent case study examining the impact of the pandemic on early childhood education experiences [15]. In this study, the main concern teachers had during the lockdown was related to their personal life, which included the health and safety of themselves and their families. Specifically, they were worried about getting sick and infecting others within proximity [15].

Participants reported working in overcrowded classrooms that made it difficult to maintain social distancing. Other reports described similar challenges following COVID-19 protocols, such as social distancing and wearing personal protective equipment (PPE), that increased perceptions of vulnerability to COVID-19 infection [24]. Evidence supports the concern that poor air conditioning and inadequate classroom ventilation can increase the spread of the virus [23]. In another study, overcrowded classrooms during the COVID-19 pandemic were linked to increased perceived vulnerability to COVID-19 [24].

There was an increase in the workload and work demands of education workers during the COVID-19 lockdown. Working from home required more effort from teachers as they planned their lessons using ICT to deliver content to students. Although there was an increase in work demands and workload, the expectations to meet these demands remained the same. In our study, education workers were working past their regular work hours to keep up with their work and continue delivering education that met the academic standards of the schools. Previous findings suggest that such an increase in workloads can lead to emotional exhaustion [25]. Simultaneously, many teachers with children had to tend to the needs of their family. Our findings suggest that these added responsibilities made it difficult to maintain a work–life balance. Based on previous studies, researchers found that working from home can be a source of conflict between work and private life [26,27]. Separating work life and private life is difficult when there are no physical boundaries reminding individuals to leave their work behind after the end of a workday. Cowden et al. (2020) found that one source of stress for educators was finding the appropriate space to work. The inability to differentiate working hours from leisure or rest can lead to interpersonal conflicts, a phenomenon which has been linked to emotional exhaustion [28].

Participants report a lack of communication from governments and school administration regarding important changes to COVID-19 policies. Education workers were not receiving information about how to proceed with their work, creating frustration and stress in the workplace. According to previous findings, initiating effective and immediate communication is crucial during natural disasters and pandemics [6]. To reduce feelings of isolation, some participants have sought out support from friends and colleagues. Researchers have found a negative relationship between perceived social support and work–family conflict [29].

Coping strategies have been adopted to help alleviate some of the stress experienced by education workers. Participants reported taking short leaves of absence from work to help maintain their well-being and reduce their feelings of stress. One study found that the stress teachers experience results from a lack of support from administration, increased workloads, lack of resources, and isolation [30]. Similarly, participants in our study reported experiencing these issues at the workplace. In this same study, researchers found that teachers who experience these stressors are more likely to take absences [30]. In comparison to men, women were more likely to take absences, especially if they were caring for sick children [30]. Physical activities and mindfulness activities helped individuals cope with stress. Previous studies found that regular physical activity engagement is an effective approach to alleviate stress and job strain [31,32]. One study examined teachers’ symptoms of stress and the coping strategies used to alleviate these symptoms and found that exercise was the only effective coping strategy [31].

There are important lessons to be learned from education workers’ experiences during a pandemic. They reported issues they faced while teaching online as well as when returning to work. One of the issues was the lack of resources provided by the school and school boards. Education workers were not given training on the use of technology and were forced to learn how to create their own content for their classrooms. The participants suggested having premade lessons for teachers to use in their classrooms to help reduce the stress and workload for employees. Furthermore, better computer equipment is important for teachers working from home. In Ferguson et al.’s (2022) study, teachers reported their lack of resources as a major stressor [30]. There was not enough funding for teachers, and there was a lack of physical resources, such as laptops and tablets, used for teaching. Similarly, our findings suggest that teachers also did not have enough resources to complete their work tasks effectively.

The findings of this study hold practical implications for governments and school leaders. Governments should consider the policies implemented during emergency situations. Before reopening schools and returning to work, education workers should receive training in implementing safe practices in the classroom to minimize the impact of emergency situations such as COVID-19. Developing a safe work environment with policies that protect the well-being of staff members and students is key to maintaining an effective learning environment despite the changes made to the physical aspect of education. Providing education workers with adequate resources and greater agency can enhance job satisfaction and commitment to the teaching profession [24]. Schools should invest in more facilities and equipment for distance learning, such as having more laptops and better Internet access, to help with the online delivery of education [15,33]. Furthermore, having technology coaches can help teachers learn and solve technological issues. Training in the use of various technologies may improve job performance and reduce the negative effects of low ICT skills [15,21]. Overall, improving the ICT skills of employees can reduce the stress of teaching online and improve the working conditions for education workers. To reduce workloads, pairing up online classes together would give teachers more time to learn the technology. Supervisor support is essential for education workers, as researchers conclude that employees who receive support have less work–family conflict and are less stressed at work [34]. Moreover, expectations for education workers should be reduced to reasonable goals. The improvement of working conditions in educational settings is important, and policies implemented should focus on promoting the health and well-being of employees. In addition to improving the working conditions of education workers, educators should be consulted when making decisions that affect them. Teachers should be able to give input on how teaching can be most effectively done in these unique conditions.

There were some limitations in this study. First, the current study is cross-sectional in design and illustrates the experiences of education workers at an instance in time. The nature of the study’s design does not allow for a causal relationship to be established. Although the study identified the stressors education workers experienced since returning to work, longitudinal research is required to understand the severity of these stressors over extended periods. Researchers should consider examining the effects of a post-COVID-19 environment on education. Furthermore, the scope of the paper does not provide an in-depth investigation of the experiences of education workers during the pandemic. Extensive research on the perceived impact of COVID-19 on education can provide a better understanding of the stressors identified in this study. Given the nature of the study, the intention of the findings was not to be generalizable but to shed light on the experiences of education workers during a global crisis.

## 5. Conclusions

Education workers have important roles in society as leaders and teachers for students. It is important to examine the impact of the pandemic on the stressors experienced by these employees. Our findings highlight the challenges that teachers have faced during online teaching and working from home, as well as the stressors they faced since returning to work. Common stressors were identified and should be considered when creating policies and programs for education workers. Education workers have been burdened by the pressures of maintaining academic standards and are challenged to solve issues posed by COVID-19 as they get little guidance and support from institutions. Policymakers and school administrators are responsible for promoting and protecting the well-being of employees. Some improvements to be made include having greater flexibility with work expectations, more training opportunities, and more support. These improvements will not only lessen the stress experienced by education workers but will also help to establish better working conditions in the workplace.

## Figures and Tables

**Table 1:** Characteristics of participants.

Characteristic	n	%
**Age**		
(min. = 18.0, max. = 81.0;		
M = 44.82; SD = 9.163)		
**Gender**		
Man	413	17.4
Woman	1928	81.1
Nonbinary or other	9	0.5
Choose not to answer	25	1.1
Missing	3	0.1
**Marital Status**		
Married/common law	1794	75.4
Separated/divorced/widowed	196	8.2
Single	333	14
Choose not to answer	49	2.1
Missing	6	0.3
**Job Classification**		
Teacher (including special education)	1995	83.9
Occasional teacher/substitute teacher	63	2.6
Computer/technician/IT	4	0.2
Clerical/office	43	1.8
Education assistant	105	4.4
Maintenance/custodial	2	0.1
Early childhood education/child and youth counselors	87	3.7
Psychological staff/social worker/speech and language pathologist	31	1.3
Other	44	1.9
Missing	4	0.2
**Work Schedule**		
Permanent full-time	2131	89.6
Permanent part-time	94	4
Temporary full-time	105	4.4
Temporary part-time	47	2
Missing	1	0.04

**Table 2:** Emerged Themes and Exemplars.

Theme	Exemplars
Challenges with service provision and using technology due to lack of training and limited resources	Difficulties adjusting to the delivery format Being forced into learning new technology Limited training opportunities
Issues with separating work and private life during the work-from-home period, creating a disruption in work–life balance	No work–life balance
Difficulties managing the return to physical classrooms resulting from a lack of clear communication and direction from the government and school administration	Conflicting information being spread Lack of communication from superiors
Fear of contracting the virus due to insufficient health/COVID-19 protocols	Colleagues not taking COVID rules seriously Failure to implement COVID protocols Overcrowded classrooms
Increase in work demands following the return to physical classrooms and the implementation of the hybrid learning model and quadmesters	Increased work hours Keeping up with work expectations Using the hybrid learning model within classrooms Quadmester schedules in high schools
Various coping strategies developed to deal with the stressors of working during the COVID-19 pandemic	Taking a leave of absence Physical activities and meditation Maintaining social relations
Lessons to be learned from working amid a global pandemic and improving the workplace in case of future emergencies	Providing more resources Giving employees more training Work from home arrangement

## Data Availability

Data presented in this study are not readily available due to ethical restrictions. Reasonable requests to access the data can be made to the corresponding author.
